# Clinical pitfalls: a painful nail enlargement

**DOI:** 10.1111/j.1468-3083.2007.02465.x

**Published:** 2008-06

**Authors:** C Cantisani, E Cigna, DM Miller, V Cantisani, F Solivetti, GM Andreoli, N Scuderi, S Calvieri

**Affiliations:** † Department of Dermatology and Plastic Surgery, University ‘La Sapienza’ of Rome Rome, Italy; ‡ Department of Radiological Science, University ‘La Sapienza’ of Rome Rome, Italy; § Department of Dermatology, Harvard Medical School Brigham and Women's Hospital, Cutaneous Oncology Dana Farber Cancer Institute, Boston VA Medical System Boston, Massachusetts; ¶ I.R.C.C.S. Ospitale Dermosifilopatico di Santa Maria e San Gallicano Istituti Fisioterapici Ospitalieri – I.F.O. Via Elio Chianesi s.n.c. – Roma – C.a.p. 00191

Editor

Alterations in the structure and appearance of the nail unit may be seen in association with a variety of dermatological disorders.^1^ Because of the limited number of reaction patterns associated with the nail unit, many of the changes that are seen, such as onycholysis, pitting and subungual hyperkeratosis, are not specific. However, certain clinical findings, especially when present in combination, may be highly suggestive of a specific diagnosis or may assist in limiting the differential diagnosis.

A 63-year-old woman was referred for the evaluation of a painful enlargement and severe third left finger nail deformity of 2 months’ duration. The lesion started at the dorsal side of the proximal nail fold and migrated distally toward the hyponychium.

**fig. 1:**
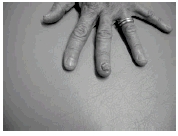
Advanced subungual hyperkeratosis resulting in uplifting of the nail plate with distal nail bed, ‘oil spot’ involvement of nearly the entire nail bed and hyponychial involvement.

Clinically, there was a greenish-blue discoloration associated to severe onychodystrophy, massive subungual hyperkeratosis causing uplifting of the nail plate and partially onycholysis at the distal phalanx (fig. 1).

The woman was otherwise in good general health, and there was no involvement of other skin areas. A fungal infection of the area was excluded by examination of a potassium hydroxide preparation. Complete general physical and mucocutaneous examination was done. Laboratory evaluation was within normal limits. The patient refused the nail biopsy. The risk of scarring and injuring the nail matrix was unacceptable to her.

**fig. 2:**
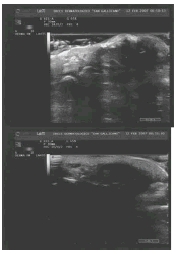
Real-time US using 13-MHZ linear probe; sonographic longitudinal section of the distal interphalangeal joint showed well-defined thickness of the nail matrix and nail bed, with slight eye catching signal at power Doppler, together with superficial soft tissue thickening at the distal phalanx.

Ultrasonography (US; fig. 2) with very high frequency transducers (Esaote AU-4 Idea Biomedica, Genoa, Italy) of the distal interphalangeal joint showed well-defined thickness of the nail matrix and nail bed. Hand X-ray film revealed acral lamellar periostitis; both were suggestive of the diagnosis of nail psoriasis. Keratolytic agents and topical Calcipotriol/betamethasone dipropionate were administered. After 2 weeks of treatment, there was a clear improvement confirming the clinical and imaging diagnosis.

Nail psoriasis can create diagnostic difficulties and may remain undiagnosed for a long time. It occurs in 10% to 50% of psoriasis patients, particularly those with arthritis, and about 1% to 5% of patients manifest nail changes alone.^2^

This is the category of patients in whom the clinician faces a formidable diagnostic challenge because psoriatic nail disease resembles other causes of dystrophic nails. Nail morphology in psoriasis depends upon the anatomical location of the disease process.^3^

The chief complaint of patients with psoriasis is the unsightliness of nails. They also battle lowered self-esteem, feelings of being socially isolated, and the stress associated with the unpredictable intervals of flaring disease and spontaneous remissions leading to significant disfigurement and disability. It is an often persistent disease and frequently refractory to treatment. Topical treatments are barely effective, and possible side-effects of systemic treatments limit their usefulness in uncomplicated nail psoriasis.^4^ The diagnosis of nail psoriasis is usually straightforward when characteristic nail findings coexist with cutaneous psoriasis.^5^ Diagnostic difficulty arises when nail disease occurs as an isolated finding. Ungueal psoriasis and onychomycosis, especially distal subungual onychomycosis, are often indistinguishable by clinical examination alone.

In our patient, the personal and family history, clinical examination, the X-ray film, dermatological sonography and a strict clinical follow-up led us to make the right diagnosis, thereby avoiding the biopsy. Dermatological sonography of soft tissues should be done more widely for the evaluation of nail psoriasis.^6^ This art requires a great deal of training and experience to be practical, and sometimes, it can substitute the microscopic examination.^7^,^8^ Advancements in technology will improve the correlation of clinical findings with high frequency ultrasound findings in the assessment of several skin diseases.
